# Exploration of the Brain in Rest: Resting-State Functional MRI Abnormalities in Patients with Classic Galactosemia

**DOI:** 10.1038/s41598-017-09242-w

**Published:** 2017-08-22

**Authors:** Britt van Erven, Bernadette M. Jansma, M. Estela Rubio-Gozalbo, Inge Timmers

**Affiliations:** 1 Department of Pediatrics and Department of Clinical Genetics, Maastricht UMC+, Maastricht, The Netherlands; 2 Maastricht Brain Imaging Center (M-BIC), Maastricht, The Netherlands; 3 Faculty of Humanities and Sciences, Maastricht University, Maastricht, The Netherlands; 4 Department of Cognitive Neuroscience, Maastricht University, Maastricht, The Netherlands

## Abstract

Patients with classic galactosemia, a genetic metabolic disorder, encounter cognitive impairments, including motor (speech), language, and memory deficits. We used functional magnetic resonance imaging to evaluate spontaneous functional connectivity during rest to investigate potential abnormalities in neural networks. We characterized networks using seed-based correlation analysis in 13 adolescent patients and 13 matched controls. Results point towards alterations in several networks, including well-known resting-state networks (e.g. default mode, salience, visual network). Particularly, patients showed alterations in networks encompassing medial prefrontal cortex, parietal lobule and (pre)cuneus, involved in spatial orientation and attention. Furthermore, altered connectivity of networks including the insula and superior frontal gyrus -important for sensory-motor integration and motor (speech) planning- was demonstrated. Lastly, abnormalities were found in networks involving occipital regions, linked to visuospatial capacities and working memory. Importantly, across several seeds, altered functional connectivity to the superior frontal cortex, anterior insula, parietal lobule and the (pre)cuneus was observed in patients, suggesting special importance of these brain regions. Moreover, these alterations correlated with neurocognitive test results, supporting a relation with the clinical phenotype. Our findings contribute to improved characterization of brain impairments in classic galactosemia and provide directions for further investigations.

## Introduction

The brain represents one of the target organs of damage in classic galactosemia, an inborn error of galactose metabolism, resulting in chronic impairments with significant impact on quality of life and general performance^1–3^. Patients with classic galactosemia suffer from a profound deficiency of galactose-1-phosphate uridylyltransferase (GALT) due to mutations in the *GALT* gene, and affected newborns present with a severe toxicity syndrome upon exposure to galactose in milk. Immediate initiation of galactose-restriction is life-saving, but fails to prevent the development of burdensome brain impairments, including lower intelligence^4–8^, language production and speech (motor) problems^9–13^, slower information processing and memory deficits^4, 14^, social difficulties^15^ and psychiatric conditions^16^. Furthermore, neurological sequelae are regularly reported, such as tremors, dystonia, coordination and balance disturbances, and ataxia^17, 18^.

The pathogenic basis of these debilitations is puzzling and there are an increasing number of studies providing evidence for structural changes in white and grey matter, as well as alterations at a functional level^17, 19–29^. Using innovative diffusion-weighted magnetic resonance imaging (MRI) analysis techniques, evidence for abnormal white matter microstructure in bilateral anterior tracts (reduced neurite density) and the left hemisphere (increased dispersion of neurites) was provided^28^, which strengthens the hypothesis of aberrant myelin composition, possibly resulting from deficient galactocerebroside formation due to aberrant glycosylation^22, 26^. Additionally, several studies demonstrated cerebral or cerebellar atrophy and decreased tissue density of grey matter^17, 19, 20, 22, 23, 25, 26, 29^. This neuronal loss has been speculated to result from direct toxicity of galactose and its metabolites or aberrant glycosylation^26^. In addition to reduced grey matter density, Timmers *et al*. also observed increased grey matter density in the inferior frontal and medial prefrontal cortex, which could reflect compensation for problematic motor and cognitive functions (memory, language) or abnormal maturation^29^.

At a functional level, Dubroff *et al*. used [18 F]fluorodeoxyglucose (FDG) positron emission tomography (PET) to evaluate glucose metabolism in patients^21^. Decreased metabolism was found in the occipital region, orbital frontal lobes, sensorimotor areas, cerebellum, superior temporal lobes, and the mid and superior parietal regions. Increased metabolism was demonstrated in bilateral anterior cingulate cortex (ACC) and temporal poles, as well as in basal ganglia. The findings in ACC and basal ganglia were interpreted as showing potential compensation for problematic motor functions.

Recently, a task-based functional MRI (fMRI) study by our group, wherein patients carried out a language production task, was the first to point towards abnormal language-related brain networks in this disorder^27^. Evaluation of neural activity demonstrated that patients recruited different and more extensive brain regions during language production as compared to controls. The left inferior frontal gyrus (IFG), the right insula and the left pre-supplementary motor area (pre-SMA) were subsequently selected as seeds for a seed-based functional connectivity analysis, which revealed variations in functional connectivity in comparison to controls.

Resting-state fMRI studies, in which neuronal connectivity in absence of a specific stimulus or task is assessed, are increasingly conducted within multiple fields of neuroscience to study the organization of core processing systems of the brain. The rationale behind these studies is that the brain is always active and shows spontaneous neuronal activity even during rest. Brain regions that show synchronous neuronal activity are considered to be functionally connected, thereby constituting a functional network^30–33^. A commonly used approach to study functional connectivity is the seed-based correlation analysis (SCA). This method is hypothesis-driven and correlates the resting-state time courses of an *a priori* selected region of interest (ROI; i.e. seed) to the time series of the rest of the brain^30, 32, 34, 35^, wherein the seed is selected from a task-dependent activation map or anatomically based on previous literature^36^. Despite differences in data acquisition and analysis methods, multiple resting-state networks have been consistently found across functional connectivity studies, such as the default mode network (DMN)^31, 32, 37, 38^. Additionally, altered functioning of these networks has been demonstrated in participants suffering from a variety of brain disorders^39^, including neurodegenerative diseases such as Alzheimer’s disease^40–42^, as well as attention deficit hyperactivity disorder (ADHD)^43–45^ and other psychiatric conditions.

In our previous study, we focused on functional networks that are involved in an active task^27^. However, resting-state fMRI allows us to investigate the overall functional organization of brain networks, which has not been studied before in classic galactosemia. The current study therefore characterized functional networks in resting-state in patients with classic galactosemia as compared to healthy controls using a seed-based approach. Seeds were selected based on previous neuroimaging studies in this population, namely the left IFG, right insula, left pre-SMA, right and left putamen, right and left medial prefrontal cortex (mPFC), and the medial occipital region^21, 27, 29^. Findings were correlated to available behavioral outcome measures as well, in order to confirm potential relations between functional networks and behavior^46^. For this, measures of functional connectivity of brain regions of interest were correlated to neurocognitive test results. Our aim was to elucidate affected brain networks in patients with this disease to improve the characterization of brain impairments encountered by these patients.

## Results

### Group differences in seed-based correlation analysis

#### Medial prefrontal cortex

**Figure 1:**
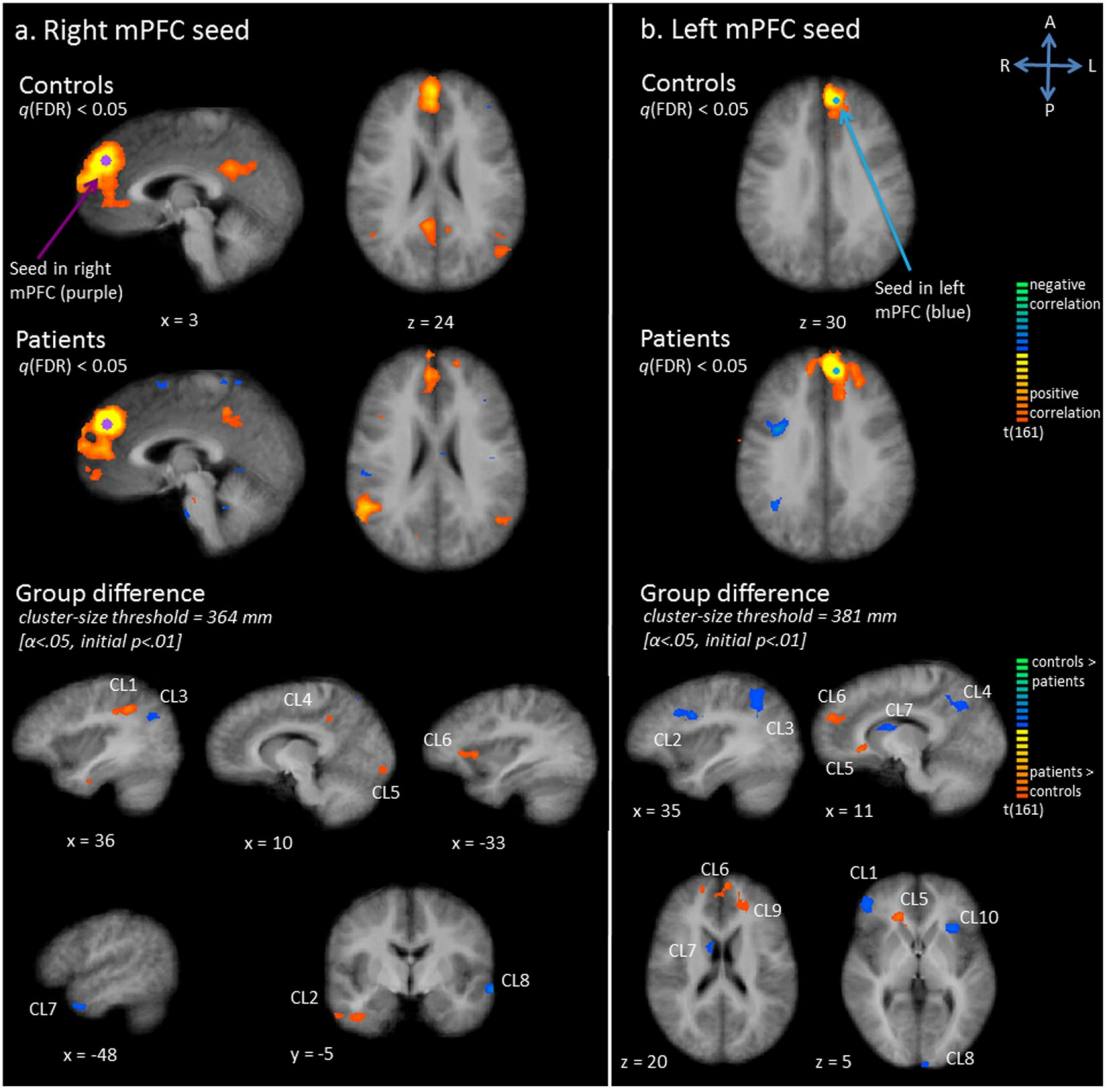
Functional connectivity (Fc) maps for the medial prefrontal cortex (mPFC) as seed; panel a: right mPFC seed, panel b: left mPFC seed. Upper and middle row: Statistical maps resulting from the seed-based correlation analysis separate per group (red indicates positive correlation, blue indicates negative correlation). Seeds are overlaid in purple (right mPFC seed) or blue (left mPFC seed). Maps are thresholded at *q*(FDR) < 0.05. Lower row: Group differences in functional connectivity per seed region (red indicates patients show increased connectivity as compared to controls, blue indicates patients show decreased connectivity as compared to controls).

In controls, activity in the right mPFC seed region correlated with left and right middle temporal gyrus (MTG) and the bilateral posterior cingulate cortex (PCC) and precuneus (Pc) (Fig. 1A). Furthermore, the bilateral ACC, left IFG and bilateral inferior temporal gyrus were correlated. In patients a generally similar, yet more extensive network was revealed on a *q*(FDR) corrected map (Fig. 1A). More extensive regions of the frontal lobe, right precuneus, PCC, right MTG and ACC were correlated with the right medial frontal seed, and in addition the right parietal region was correlated. Statistical comparison between the two groups confirmed increased connectivity with the right inferior parietal lobe (IPL) / angular gyrus, right inferior temporal lobe, right PCC/precuneus, right lingual gyrus and left anterior insula in patients, compared to controls (Fig. 1A, Table 1). Decreased connectivity in patients was demonstrated in right superior parietal lobe (SPL), left temporal pole and left middle temporal gyrus (MTG).

**Table 1:** Group differences per seed and significant correlations with neurocognitive tests. Clusters (cl.) of difference in functional connectivity between controls (c) and patients (p) specified per seed. Size and peak Talairach coordinates of each cluster are presented. Furthermore, significant correlations between clusters and neurocognitive test results are shown (for patient group only); clusters surviving a Bonferroni correction are marked (*). Tests used: Rey Immediate T-score for visuospatial working memory^74^, Digit span for verbal working memory^75^, Bourdon-Vos mean Reaction Time for sustained attention^76^, voice onset times for language production^12^.

	Group difference	Nr of voxels	Peak Talairach coordinate			Significant correlation (patients only)
			X	Y	Z	
Right Insula – Cl. 1 (right SFG)	p > c	921	24	50	16	Voice onset times (*r* = 0.62; *p* = 0.03)
Occipital region – Cl. 1 (left cuneus)	c > p	2460	−12	−97	7	Verbal working memory (*r* = −0.61; *p* = 0.04)
Left putamen – Cl. 1 (left SPL)	c > p	448	−22	−67	61	
Right putamen – Cl. 1 (right MTG)	c > p	445	51	5	−14	
Right putamen – Cl. 2 (right thalamus)	p > c	2167	12	−25	10	
Right putamen – Cl. 3 (right lentiform nucleus)	c > p	724	21	17	−2	
Right putamen – Cl. 4 (left PCC)	c > p	995	−9	−37	7	
Left mPFC – Cl. 1 (right IFG)	c > p	812	45	41	4	
Left mPFC – Cl. 2 (right PrG)	c > p	1509	33	5	31	
Left mPFC – Cl. 3 (right SPL)	c > p	3119	30	−46	46	Voice onset times (*r* = −0.60; *p* = 0.04)
Left mPFC – Cl. 4 (right precuneus)	c > p	1876	18	−52	40	Voice onset times (*r* = −0.71; *p* = 0.009) *
Left mPFC – Cl. 5 (right ACC)	p > c	1232	15	32	7	
Left mPFC – Cl. 6 (bilateral mPFC)	p > c	2724	−3	50	28	
Left mPFC – Cl. 7 (right caudate body)	c > p	444	12	2	19	
Left mPFC – Cl. 8 (left cuneus)	c > p	600	−3	−91	13	
Left mPFC – Cl. 9 (left SFG)	p > c	585	−21	44	22	
Left mPFC – Cl. 10 (left anterior insula)	p > c	550	−27	23	4	Visuospatial working memory (*r* = −0.67; *p* = 0.02)
Right mPFC – Cl. 1 (right IPL)	p > c	8293	39	−40	37	
Right mPFC – Cl. 2 (right temporal lobe)	c > p	711	39	−7	−26	Voice onset times (*r* = 0.58; *p* = 0.05)
Right mPFC – Cl. 3 (right SPL)	c > p	1459	24	−67	59	
Right mPFC – Cl. 4 (right PCC/precuneus)	p > c	508	15	−43	37	
Right mPFC – Cl. 5 (right occipital lobe)	p > c	465	9	−88	−8	
Right mPFC – Cl. 6 (left anterior insula)	p > c	449	−33	29	7	Visuospatial working memory (*r* = 0.70; *p* = 0.01) *
Right mPFC – Cl. 7 (left temporal lobe)	p > c	433	−48	5	−26	
Right mPFC – Cl. 8 (left MTG)	c > p	487	−60	−7	−5	

Connectivity maps showed that the left mPFC seed was functionally connected with the surrounding medial and superior frontal area, as well as with the dorsal ACC (bilaterally) in the control group (Fig. 1B). In patients, activity of the seed correlated with more extensive parts of the left and right frontal regions, as well as with the left middle temporal lobe and both parietal lobes (right > left) (Fig. 1B). Statistical tests corroborated increased functional connectivity in patients between the seed and right ACC, bilateral mPFC / superior frontal gyrus (SFG), and left SFG. (Fig. 1B, Table 1). Decreased functional connectivity was found with right IFG, right PrG, right SPL, right precuneus, right caudate, as well as left cuneus and left anterior insula.

### Putamen

**Figure 2:**
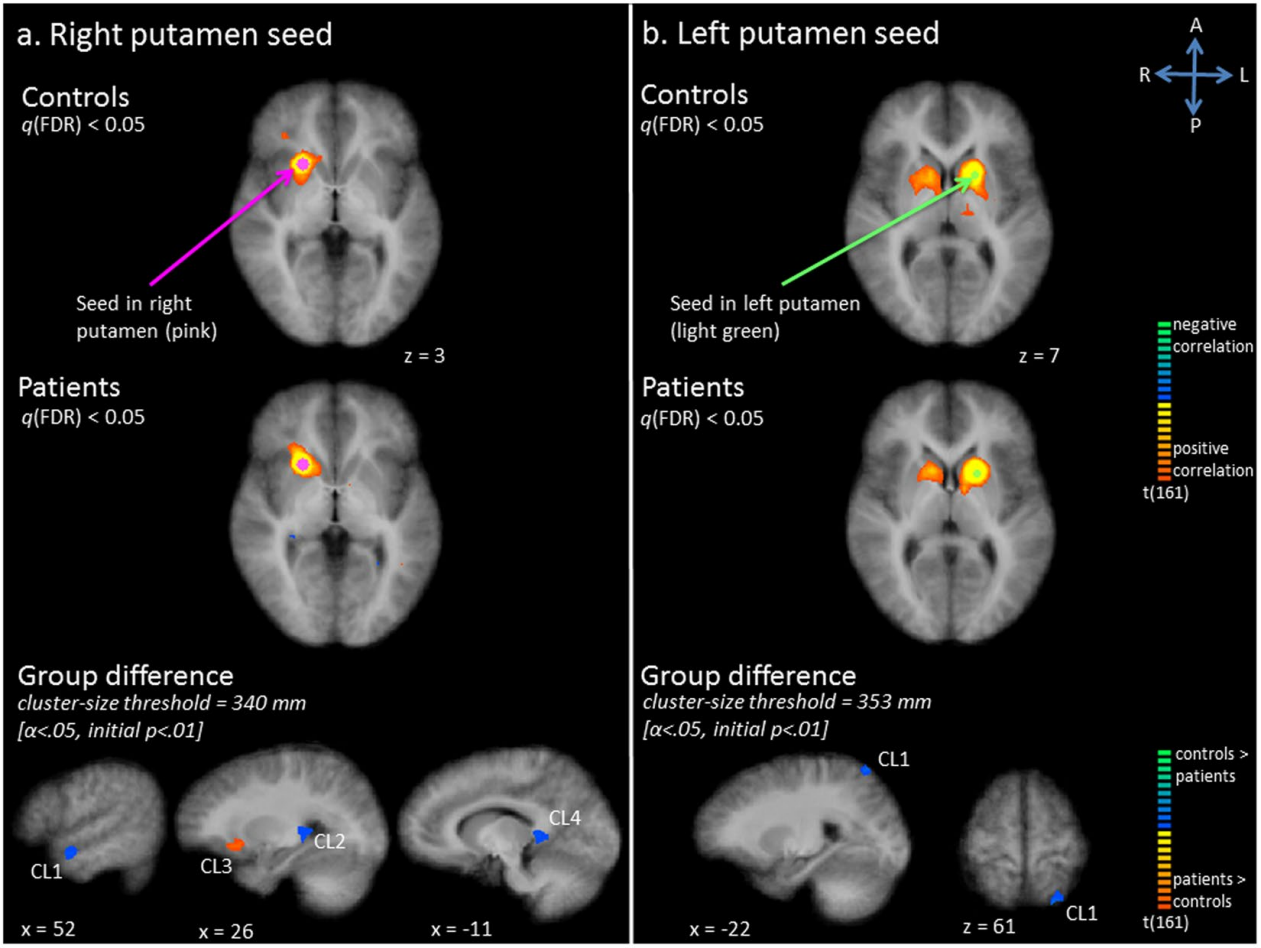
Functional connectivity (Fc) maps for the putamen seed; panel a: right putamen seed, panel b: left putamen seed. Upper and middle row: Statistical maps resulting from the seed-based correlation analysis separate per group. Seeds are overlaid in pink (right putamen seed) or light green (left putamen seed). Maps are thresholded at *q*(FDR) < 0.05. Lower row: Group differences in functional connectivity per seed region (red indicates patients show increased connectivity as compared to controls, blue indicates patients show decreased connectivity as compared to controls).

The seed in the right putamen showed temporal correlation with the head of the right nucleus caudatus and right thalamus, as well as with a small region of the right IFG (Fig. 2A). In the patient group, a more extensive region of the right IFG was correlated. Furthermore, additional correlations were seen with right insula, right ACC and right MCC (Fig. 2A). Statistical comparisons between patients and controls demonstrated increased functional connectivity with right orbito-frontal cortex, extending to the putamen in the patient group (Fig. 2A, Table 1). Decreased functional connectivity was found in right MTG, right thalamus, and left PCC.

In controls, activity in the left putamen seed correlated with activity in the left caudate nucleus, left thalamus, right caudate nucleus and right putamen (Fig. 2B). A similar network was shown in patients. However, additional correlations with the left ACC and right thalamus were seen on the *q*(FDR) corrected map (Fig. 2B). The statistical comparison only revealed decreased connectivity with left SPL in patients compared to controls (Fig. 2B, Table 1).

### Left inferior frontal gyrus

**Figure 3:**
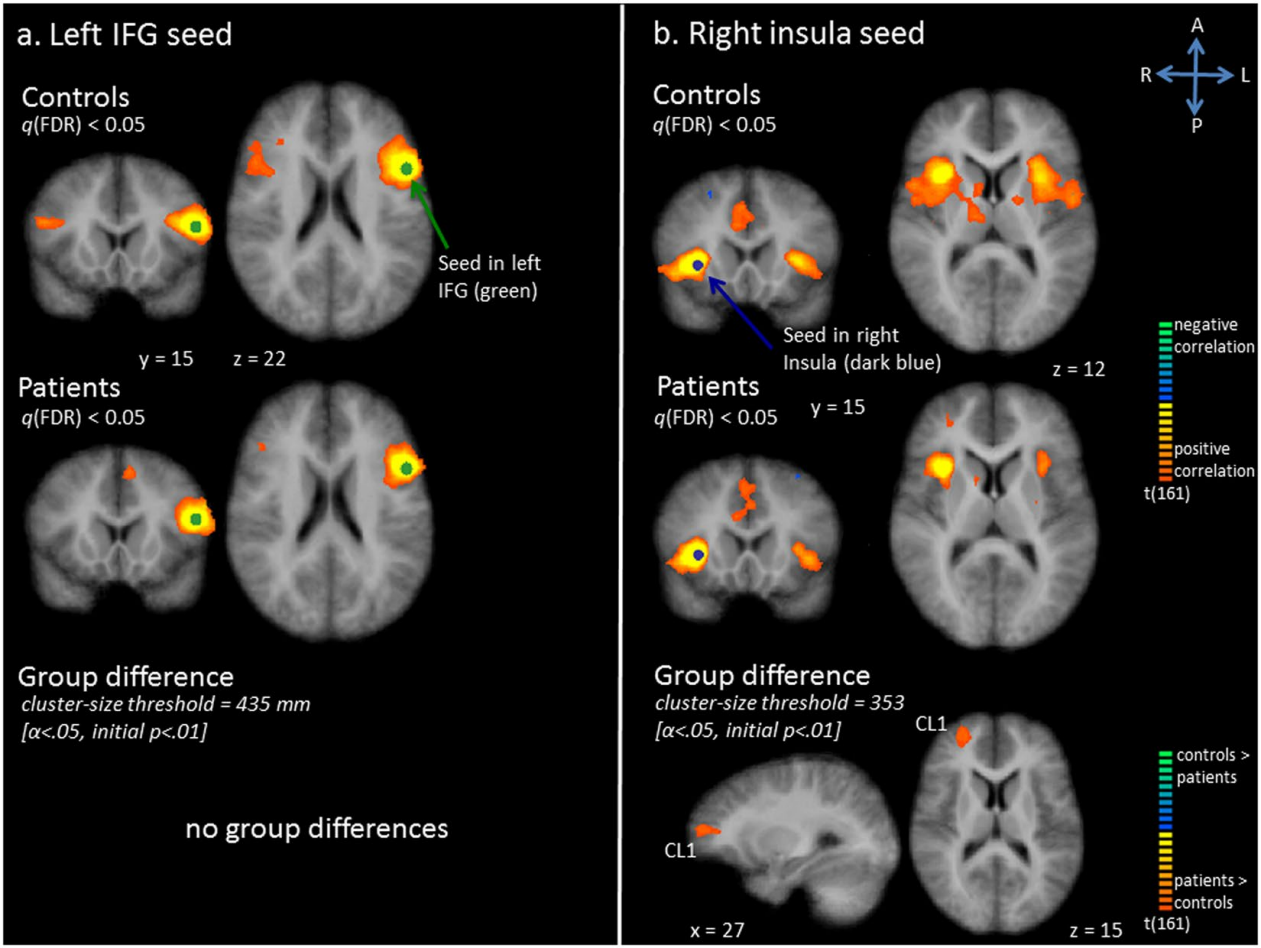
Functional connectivity (Fc) maps for the left inferior frontal gyrus (IFG) seed (panel a) and right insula seed (panel b). Upper and middle row: Statistical maps resulting from the seed-based correlation analysis separate per group. Seeds are overlaid in green (left IFG seed) or dark blue (right insula seed). Maps are thresholded at *q*(FDR) < 0.05. Lower row: Group differences in functional connectivity per seed region (red indicates patients show increased connectivity as compared to controls, blue indicates patients show decreased connectivity as compared to controls).

The seed in the left IFG showed temporal correlation with the left mPFC, left PrG, bilateral MTG, right IFG, left IPL and SPL, left supramarginal gyrus, left precuneus and left angular gyrus in controls (Fig. 3A). The *q*(FDR) corrected map revealed a less extensive network in patients, with less extensive correlation between the seed and its surrounding area, the contralateral side, and the left parietal lobule (Fig. 3A). However, statistical tests did not show any statistically significant group differences.

### Right insula

In controls, activity in the right insula seed showed correlation with activity in right pre-SMA, bilateral PrG, bilateral PoG, bilateral ACC/MCC, right MFG, bilateral IFG, right nucleus caudatus, right putamen, right thalamus, right IPL, left insula and left cuneus (Fig. 3B). More extensive correlation with bilateral pre-SMA regions and the right SFG was noticed in patients, as compared with controls (Fig. 3B). Less extensive correlation was seen with right basal ganglia and thalamus, left insula, left IFG and left PoG. The statistical comparison corroborated increased connectivity with right SFG in patients, as compared to controls (Fig. 3B, Table 1).

### Left pre-supplementary motor area

**Figure 4:**
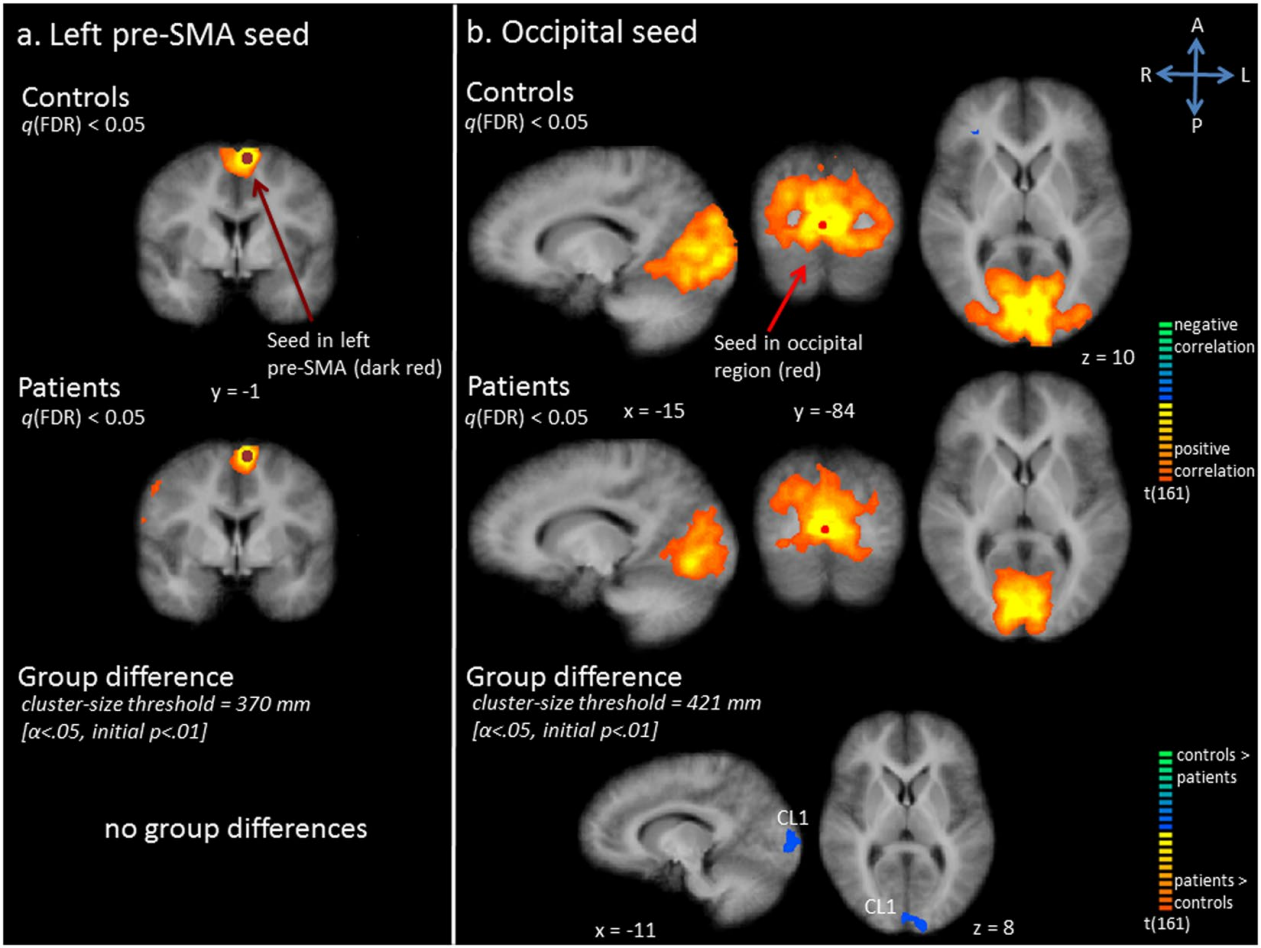
Functional connectivity (Fc) maps for left pre-supplementary motor area (SMA) seed (panel a) and occipital seed (panel b). Upper and middle row: Statistical maps resulting from the seed-based correlation analysis separate per group. Seeds are overlaid in dark red (left pre-SMA seed) or red (occipital seed). Maps are thresholded at *q*(FDR) < 0.05. Lower row: Group differences in functional connectivity per seed region (red indicates patients show increased connectivity as compared to controls, blue indicates patients show decreased connectivity as compared to controls).

Connectivity maps showed that the left pre-SMA seed was functionally connected with bilateral (pre-)SMA and premotor cortices and the left PrG in the control group (Fig. 4A). A similar network was seen in patients. However, several additional regions were correlated, including the right PrG, the left mPFC and left SFG (Fig. 4A). Statistical comparison failed to show any significant group differences.

### Occipital region

Activity in the medial occipital region seed correlated with activity in bilateral middle occipital gyrus, bilateral lingual gyrus, extrastriate cortex, bilateral cuneus, the right precuneus, the right and left PrG in controls (Fig. 4B). In the control group bilateral PrG, right pre-SMA and cerebellum were also correlated. In patients, less extensive correlation was seen with the occipital region surrounding the seed, especially bilateral middle occipital gyri and cuneus, as well as with right precuneus, cerebellum and motor areas (Fig. 4B). Statistical tests confirmed decreased functional connectivity in patients with regard to left cuneus (Fig. 4B, Table 1).

### Correlations with neurocognitive tests

**Figure 5:**
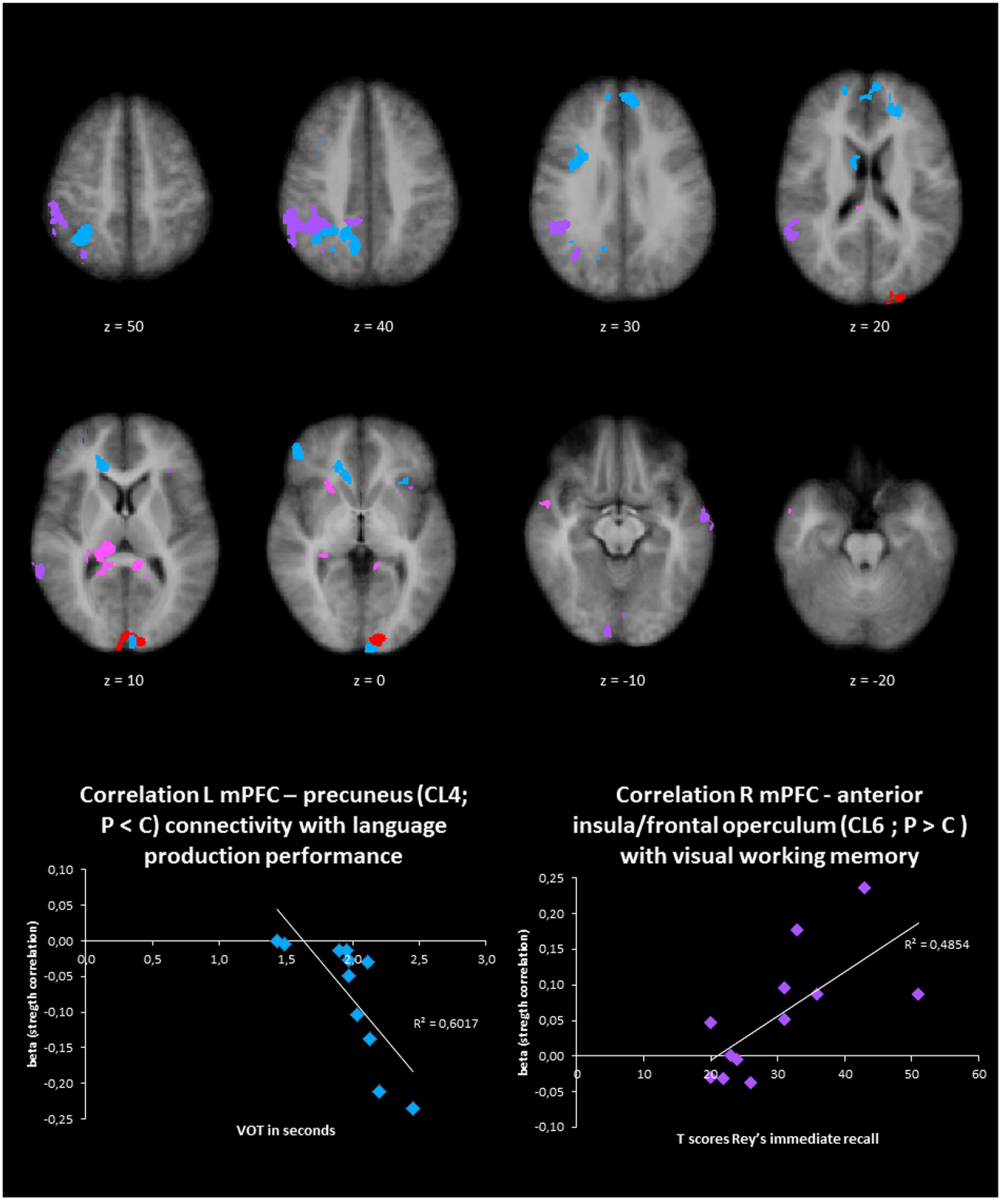
Clusters (VOI) of group differences. Upper part: Overview of clusters (VOI) of group differences, specified per seed (see Table 1). Lower part: Scatterplots of clusters with significant correlations to voice onset times^12^ and visuospatial working memory (Rey Immediate Recall T-score^74﻿^), surviving a Bonferroni correction.

A significant correlation between the patients’ beta values (representing the functional connectivity with the seed region) and results of neurocognitive tests was found for eight clusters (Table 1, Fig. 5A). Several seed-cluster connectivity measures correlated with voice onset times (VOTs) from a language production task. Functional connectivity between the right insula and the right SFG (cluster 1) was positively correlated with VOTs (*r* = 0.62, *p* = 0.03). This cluster showed increased functional connectivity in the patient group as compared to controls, which altogether suggests that increased connectivity correlates with lower performance (slower VOTs) in patients. Functional connectivity between the left mPFC and both right SPL and right precuneus were negatively correlated with the VOTs (*r* = −0.60, *p* = 0.04; *r* = −0.71, *p* = 0.009, surviving Bonferroni correction). These clusters showed decreased functional connectivity in patients as compared to controls, which altogether suggests that decreased connectivity correlates with lower performance (slower VOTs) on language production in the patient group. Lastly, functional connectivity between the right mPFC and right temporal lobe was positively correlated with VOTs (*r* = 0.58, *p* = 0.05). This cluster showed increased functional connectivity in the patient group as compared to controls, which altogether suggests that increased connectivity correlates with lower language performance (slower VOTs) in patients.

Correlations between functional connectivity patterns and visuospatial working memory were observed as well, both involving the left anterior insula (cluster 10 for the left mPFC seed, cluster 6 for the right mPFC seed). Functional connectivity between the left mPFC and left anterior insula was negatively correlated with visuospatial working memory (*r* = −0.67, *p* = 0.02). This cluster showed decreased functional connectivity in patients as compared to controls, which altogether suggests that decreased connectivity correlates with better performance in patients. On the other hand, functional connectivity between the right mPFC and the left anterior insula was positively correlated with visuospatial working memory (*r* = 0.70, *p* = 0.01, surviving Bonferroni correction). This cluster showed increased functional connectivity in patients as compared to controls), which altogether suggests than an increased connectivity correlates with higher performance on visuospatial working memory in the patient group.

Finally, verbal working memory was correlated to functional connectivity patterns too. Functional connectivity between the occipital region and left cuneus (cluster 1) was negatively correlated with verbal working memory (*r* = −0.61, *p* = 0.04): decreased functional connectivity correlated with better performance in patients. As this cluster showed decreased connectivity in patients as compared to controls, this suggests that more dissimilar connectivity pattern to controls are associated with better performance in patients.

## Discussion

In the current study, we explored intrinsic functional networks by investigating spontaneous correlations in functional activity during rest in patients with classic galactosemia. The right and left mPFC, right and left putamen, left IFG, right insula, left pre-SMA and occipital region were selected as seeds for a SCA, based on previous studies indicating altered structure, function and/or metabolic activity of these brain regions^21, 27, 29^. Results from the SCA point towards several differences in these resting-state functional networks between patients with classic galactosemia and age- and gender-matched controls. Importantly, group differences per seed show substantial overlap. Independent of the seed region, differences in connectivity across groups were observed within the superior frontal cortex, parietal cortex (including SPL, PCC, precuneus), cuneus and anterior insula. These results indicate a special role of these brain regions within the altered brain connectivity in this patient group (Fig. 5).

Previous studies consistently identified a set of resting-state networks across different participants and analysis strategies^31, 37, 38^. Some of the *a priori* selected seeds for our study constitute key components of these resting-state networks. In line with this, our SCA points out these well-described networks.

The mPFC is part of the DMN, which has been of major interest over the past years. This network also comprises the PCC/precuneus and parietal cortex (inferior parietal / angular cortex), and has been associated with introspective processes such as attention and consciousness, integration of cognitive and emotional information, and episodic memory^31, 32, 38, 47, 48^. Using the mPFC as a seed, the DMN was observed in both patients and controls, yet multiple alterations in functional connectivity within this network were revealed in patients as compared to controls. The right and left mPFC seed analyses indicated decreased connectivity with the SPL. Assuming that decreased connectivity relates to impaired cognitive function, these altered connectivity patterns of prefrontal executive functions and SPL may relate to problems in visuospatial orientation, as the SPL has a relevant role in spatial orientation, locating objects and body parts in space, and visuospatial attention^49, 50^. This interpretation is in line with the below average scores on visuomotor/-perceptual/-spatial functioning tests and working memory assessments found in patients with classic galactosemia^4, 5, 7, 14, 51, 52^. The decreased connectivity between left mPFC and both SPL and precuneus correlated with poorer language production performance in patients. The right mPFC seed further showed increased connectivity patterns with IPL and precuneus. Assuming that increased connectivity relates to compensatory functions, the observed increased connectivity of mPFC and IPL could be related to elevation of attention, as the IPL is a relevant region in the attention network^49, 53^.

The insula constitutes a key element of the salience network. This resting-state network additionally consists of the ACC as well as other subcortical and limbic structures, and is suggested to integrate external sensory information with internal emotional and bodily state signals in order to guide behavior, thereby engaging bottom-up detection, attention, working memory and motor systems^54–56^. The right insula seed analysis revealed this network in both groups, yet with altered connectivity in patients as compared to controls. Aberrant connectivity between the anterior insula and SFG/mPFC was observed across multiple seed analyses in this study. A meta-analysis of functional neuroimaging data indicates that the insula provides an interface between feelings, cognition and action, and can be subdivided in three parts with distinct functional connectivity profiles^57^: dorsal anterior insula (high-level cognitive processes, e.g. task switching, inhibition, error processing, as well as motor sequencing and speech planning^56, 58^), ventral anterior insula (affective processes; e.g. perception of emotions^56^) and posterior insula (sensory-motor processes; e.g. tactile perception, as well as skeleto-body orientation^56, 59^). The altered connectivity between prefrontal regions related to executive functions and sensory-motor integration structures may relate to (compensation of) problems in the patients motor sequence planning, including motor speech production and planning difficulties^58, 60^. This would be in agreement with previously described motor (speech) disorders in classic galactosemia^9, 11, 17, 61^, as well as with the observed correlation between increased connectivity and poorer language production performance.

The visual network, encompassing a primary visual component (medial visual areas, including striate cortex) and an extrastriate component (lateral visual areas, such as extrastriate cortex and occipito-temporal regions), has been associated with processing of visual information^31, 38, 62^. We observed alterations in the occipital seed functional connectivity in the patient group. Evaluation of the occipital seed analysis showed decreased connectivity with the cuneus, which has been implicated in visuospatial attention/processing^63, 64^. Findings from the right and left putamen analyses (altered connectivity with thalamus^65^ and SPL) are also in line with this hypothesis.

Additionally, the STG and MTG, which are important for perception and production of language and speech^66^, as well as for social cognition^67, 68^, showed abnormal connectivity in patients across several analyses. Previous studies from our group already demonstrated specific abnormalities in the language network leading to lexical and syntactic planning impairments, in addition to often observed motor speech planning problems^12, 27^. The finding of an association between mPFC – temporal cortex connectivity and language production performance supports this further.

The correlations between the patients’ resting-state brain connectivity patterns and their performances on several neurocognitive tasks were used to further investigate the relation between brain networks and behavior. Note that we only selected those clusters that were different between patients and controls, and that correlations could only be explored for the patient group. Nevertheless, we observed several strong and specific correlations for several clusters, with correlations for two clusters surviving even a Bonferroni correction. The correlations should be interpreted with caution, but they might support the general functional interpretation of our resting-state analysis. The observed correlations seem to underline a brain network-behavior relation, although this needs to be confirmed in task-based fMRI studies targeting these specific neurocognitive functions. Most importantly, brain-behavior correlations were detected especially for those clusters that come back across multiple seed analyses as being divergently connected (compared to controls), namely the insula, parietal lobule and (pre)cuneus (Fig. 5), thereby supporting the suggested relevance of certain connectivity problems in these patients.

In general, these findings shed new light on the brain impairments in classic galactosemia. Future studies are needed to investigate whether the alterations observed in this study are the result of targeted damage to these specific regions/networks, or whether these structures are equally affected as other regions of the brain but show a more prominent functional impairment. Alternatively, these differences in functional connectivity might reflect compensation mechanisms to cope with cognitive difficulties. In order to provide more detailed hypotheses on this, investigations of longitudinal data and a systematic comparison of results of structural and functional brain imaging studies are needed.

The current findings are in line with earlier studies in the field of classic galactosemia. A previous tasked-evoked fMRI study from our group already demonstrated altered functional connectivity in the language network, with specific alterations at the level of the IFG, insula and pre-SMA, suggesting impaired sensory-motor integration, motor (speech) planning deficits and suboptimal communication between frontal regions and temporal/parietal regions^27^. The connectivity patterns and network alterations are in agreement with the current resting-state findings. The observed language network abnormalities are also in line with an earlier syntactic language planning study using event related potentials (ERPs), in which lexical and syntactic planning impairments were seen^12^. Results from an FDG-PET study pointed towards abnormalities of the superior temporal lobes, parietal regions, primary visual cortex, sensorimotor areas and frontal lobes^21^, brain regions that had a repeatedly altered functional connectivity in the current study. Imaging studies exploring brain structure in patients with classic galactosemia provided evidence for both white and grey matter abnormalities in line with the neurocognitive profile of patients^28, 29^ and with the here reported results. Previous investigations in classic galactosemia pointed towards cerebellar involvement as well^17, 19, 20, 22, 26^. Although this brain structure was only partially included in the functional coverage of the fMRI scans, altered functional connectivity with the cerebellum was not observed in our study. This might be explained by the presence of only mild neurological signs and symptoms in our patient sample (as cerebellar dysfunction is associated with neurologic signs), though it should be noted that these were not systematically evaluated (information derived from medical records). In addition, the *a priori* selected seeds were all cortical, resulting in neural networks that are mainly (sub)cortical. Future studies specifically tapping into cerebellar connectivity are needed to shed more light on this topic.

One of the limitations of this study is its limited sample size, making this study of explorative nature. The small cohort size hampers solid conclusions on functional connectivity and confirmation of results in a larger sample is thus warranted. Moreover, gender was not equally balanced in our test sample (females > males) and this cohort is therefore not fully representative for the classic galactosemia population. Though cognitive performance does not seem to differ between male and female patients^16^, future studies exploring potential gender variations in functional connectivity are desired. Furthermore, the current study method is based on certain assumptions and focuses on *a priori* selected seeds, deriving from previous investigations. Future resting-state fMRI studies could consider an independent component analysis approach, which analyzes the overall pattern of functional connectivity without focusing on specific regions of interest^69–71^. In addition, we correlated functional connectivity to results on neurocognitive tests in order to evaluate potential brain-behavior relations. Since correlations could only be explored for the patient group, conclusions should be drawn with caution. These findings provide the first conjectures on potential brain-behavior relationships and the hypotheses generated by the current study require further testing in more dedicated study designs.

Taken together, robust differences in functional resting-state networks between patients with classic galactosemia and age- and gender-matched controls were found. Results from our SCA point towards alterations in several networks, including some well-known resting-state networks (DMN, salience network, visual network). More specifically, abnormalities were revealed in networks encompassing the mPFC, parietal lobule and (pre)cuneus, which are involved in spatial orientation and attention. Furthermore, altered connectivity of networks including the insula and SFG, which are important for sensory-motor integration and motor (speech) planning/sequencing, was demonstrated. Lastly, abnormalities were found in resting-state networks involving the occipital region and cuneus, which might be linked to the observed impairments in visuospatial capacities and working memory. Most importantly, across several seeds, altered functional connectivity to the superior frontal cortex, anterior insula, parietal lobule and the (pre)cuneus was observed in patients, suggesting these brain regions might be of special importance and deserve more thorough investigations. In addition, these differences in network connectivity correlated with clinical test results in patients, supporting a relation between functional abnormalities and the clinical phenotype. Our findings contribute to the characterization of functional brain impairments in classic galactosemia, which is needed to create a better understanding of this enigmatic disease.

## Material and Methods

### Participants

Thirteen adolescent patients with classic galactosemia and thirteen age- and gender-matched healthy controls participated in this study. Participants were recruited as described by Timmers *et al*.^27^. Briefly, patients who participated in an earlier study of our group and aged 14 years or older were invited to participate in the current study. Classic galactosemia diagnosis was confirmed by GALT enzyme activity measurement and/or *GALT* mutation analysis. One patient and one control subject were excluded because of extensive motion during scanning and a current health condition, respectively. Characteristics of included patients and controls are presented in Table 2. Participants had no other relevant health conditions, had normal or corrected to normal vision, were native Dutch speakers, and were eligible for MRI assessment. All gave written informed consent, and in case of minors informed consent was obtained from the parents/caregivers as well. The Medical Ethical Committee of the University Hospital Maastricht/University of Maastricht (azM/UM) gave ethical approval for this study. The study was conducted in accordance with the Declaration of Helsinki.

**Table 2:** Group characteristics. 1: Median age was not significantly different between groups (F_1,22_ = 0.12, *p* = 0.73). 2: GALT enzyme activity measured at diagnosis. 3: Special education, speech therapy or motor therapy at some point in life. 4: Immediate Recall subtest of Rey Osterreith Complex Figure T-score^74^. 5: Digit Span (Forward and Backward) score^75^. 6: Bourdon-Vos mean reaction time^76^. 7: Voice onset times reflecting language production^12^.

	Patients (n = 12)	Controls (n = 12)
	Value (range)	Value (range)
Gender	Males: 3	Males: 3
	Females: 9	Females: 9
Age (years)^1^	17.4 (14.6–21.1)	17.1 (14.0–20.0)
Age at initiation of galactose-restricted diet (days)	11.0 (0–35)	
GALT activity (% of reference value)^2^	0.55 (0–1.52)	
*GALT* mutation	5x p.Q188R/p.Q188R (42%), 1x p.Q188R/p.L195P (8%), 3x p.L195P/p.K229N (25%), 2x p.W134fs/unknown (17%), 1x Unknown (8%)	
Special education (% of group)^3^	75	
Speech therapy (% of group)^3^	92	
Motor therapy (% of group)^3^	42	
Visuospatial working memory^4^	30 (<20–51)	
Verbal working memory^5^	4.25 (2–7)	
Sustained attention^6^	14.9 (11.3–20.3)	
Voice onset times^7^	2.0 (1.4–2.6)	

### Procedure

To prevent excessive motion during scanning, participants practiced lying in a dummy scanner. After this practice session, participants received explicit instructions and were placed comfortably in the MRI scanner. Resting-state data were acquired, followed by a language task. The task-evoked functional connectivity pattern analyses were described elsewhere^27^. During the resting-state scan (duration: 6 minutes) a fixation point was presented and participants were instructed to relax, not to think of something in particular and to keep their eyes open. Total duration of the experiment, including the acquisition of other data, was approximately 90 minutes. Afterwards, participants completed a short questionnaire addressing the difficulty of the session (not significantly different between patients and controls; *t* = 0.562, *df* = 24, *p* = 0.58).

### Data acquisition

Data were obtained on a 3 T Siemens MAGNETOM® Allegra head scanner using an 8-channel head coil, and a 3 T Siemens MAGNETOM® Trio whole body scanner using a 32-channel head coil (Siemens Medical System, Erlangen, Germany). Data acquisition on two different scanners was necessary as a result of irresolvable technical issues with the Allegra scanner. Four patients and four controls were scanned on this MRI scanner. Parameters of the Allegra scanner and Trio scanner were identical unless otherwise specified.

T1-weighted anatomical images were acquired using an ADNI MPRAGE sequence with 192 slices and 1mm iso-voxel resolution covering the whole brain (repetition time [TR] = 2250 ms; echo time [TE] = 2.6 ms). Functional T2*-weighted resting-state images were obtained using a standard echo-planar imaging (EPI) sequence covering the whole brain, except for cerebellum (TR = 2000 ms, TE = 30 ms, 32 slices, 180 volumes, 3.5 mm iso-voxel).

### Data pre-processing

Data were analyzed using BrainVoyager QX version 2.8.4.2645 (Brain Innovation, Maastricht, the Netherlands). For functional datasets, the first four volumes of each complete time series were discarded because of saturation effects. Pre-processing of the functional data included correction for slice time differences, 3D head motion correction (none of the six parameters differed significantly between patients and controls, *p* > 0.05 for all parameters), linear trend removal and spatial smoothing (Gaussian filter FWHM of 4 mm). Functional datasets were co-registered with anatomical data and normalized in Talairach space (3.5 mm iso-voxel). Anatomical data of all participants were averaged to create a group-based dataset.

### Statistical analyses

We conducted a SCA with the left IFG, right insula, left pre-SMA, right and left putamen, right and left mPFC, and the medial occipital region as seeds (a sphere was created surrounding the peak voxel and taken as region of interest [ROI], 257 voxels; Table 3). The six detrended 3D head motion parameters and their derivatives, head motion spikes (not significantly different between patients and controls, *t* = −0.974, *df* = 22, *p* = 0.34), the extracted mean signal from the cerebral spinal fluid (CSF) and white matter (WM), and the global signal were Z-normalized and added as variables of no interest. Though the approach remains a subject of debate^72, 73^, we decided to regress out the global signal since we are interested in localized network-specific neuronal activity, which could be obscured by unspecific global blood oxygenation level dependent (BOLD) fluctuations. Furthermore, signal oscillations at a frequency of 0.1–0.25 Hz (sine-cosine pairs) were added as confounders for low-pass filtering of the time series.

**Table 3:** Regions of interest (ROIs) used as seeds in seed-based correlation analysis. Mean Talairach coordinates (center of gravity) for seeds used in seed-based correlation analysis are presented. Abbreviations: IFG = inferior frontal gyrus, pre-SMA = pre-supplementary motor area, mPFC = medial prefrontal cortex.

	Mean X Talairach coordinate	Mean Y Talairach coordinate	Mean Z Talairach coordinate
Left IFG	−49	13	22
Left pre-SMA	−6	−2	62
Right insula	32	14	6
Right mPFC	3	45	34
Left mPFC	−8	48	28
Right putamen	21	15	1
Left putamen	−17	9	4
Occipital region	2	−81	2

The BOLD time courses of the seeds were extracted, normalized and correlated with the time series from all other brain voxels using a random-effects (RFX) group analysis. We first estimated functional connectivity for each participant separately in a multi-study RFX General Linear Model analysis (first level), which then served as input for a second-level analysis of variance (ANOVA), allowing group contrasts. Functional connectivity per group was inspected at the level of the whole cerebrum on maps corrected at *q*(FDR) < 0.05. For contrasts across groups, an initial threshold of *p* < 0.01 was chosen after which a cluster-size thresholding was performed using MonteCarlo simulations (n = 1000) to correct maps for multiple testing at the level of alpha 0.05.

### Correlations with neurocognitive tests

From clusters in which group differences were observed, beta values were extracted. In the patient group, the beta values per cluster (representing the functional connectivity of that region with the seed region) were correlated with results of neurocognitive tests (performed prior to the current study as part of a different investigation by our group^12^) using a Pearson correlation analysis. The Immediate Recall subtest of the Rey Osterreith Complex Figure (expressed as T-score) was used to evaluate visual working memory^74^. The Digit Span (Forward and Backward) measured verbal working memory^75^. The Bourdon-Vos test was used to assess sustained attention (mean reaction time [RT])^76^. Voice onset times (VOTs) from a sentence production task of a previous study in the same patient group were used as a measure of language production^12^. Correlations with a *p* value < 0.05 were considered statistically significant.
